# Bilateral radial collateral ligament rupture in a shoemaker: A case report and brief literature review

**DOI:** 10.1097/MD.0000000000020126

**Published:** 2020-05-08

**Authors:** Yueying Li, Guangzhi Wu, Shusen Cui, Zhan Zhang, Xiaosong Gu

**Affiliations:** a Department of Hand Surgery, China-Japan Union Hospital of Jilin University, Changchun, Jilin; b Key Laboratory of Neuro regeneration, Ministry of Education and Jiangsu Province, Co-innovation Center of Neuro Regeneration, Nantong University, Nantong, Jiangsu, P.R. China.

**Keywords:** bilateral, radial collateral ligament, shoemaker

## Abstract

**Introduction:**

Rupture of the radial collateral ligament (RCL) of the index metacarpophalangeal (MCP) joint is mostly related to acute local mechanical causes, which severely affect the stability of the MCP joint. However, few cases of chronic bilateral job-related RCL injury have been reported in the literature. There is no consensus on the knowledge of the disease to date. Here, we present an extremely rare case of chronic bilateral RCL injury.

**Patient concerns:**

A 58-year-old female shoemaker presented with chief complaints of swelling and pain in the radial aspect of the MCP joint of bilateral index fingers since 2 years. There was no history of acute RCL injury. The persistent pain was aggravated while gripping, pulling, buttoning, and twisting.

**Diagnosis:**

Based on the combination of physical examination, X-ray, and ultrasonic and magnetic resonance imaging, the patient was diagnosed with bilateral tear of the RCLs and joint dislocation of the index MCP joint. Eventually, intra-operative findings confirmed the diagnosis.

**Intervention:**

The patient underwent bilateral index MCP joint fusion followed by immobilization for 6 weeks. Functional therapy was started after immobilization.

**Outcomes:**

The patient's chief complaints were significantly alleviated after the operation. At the 12-month follow-up, the patient returned to a full level of activity as a shoemaker without any complications.

**Conclusion:**

Compared to acute RCL rupture of the index MCP joint, occupation may play an important role in the diagnosis of chronic RCL rupture of the index MCP joint. Our report will provide more diagnostic and treatment experience to deal with this type of injury.

## 1 Introduction

Radial collateral ligament (RCL) injuries of the index finger are rare and underreported.^[1]^ The prevalence of RCL injury is 1 in 1000 hand injuries including metacarpophalangeal (MCP) joint collateral ligament injuries.^[2]^ One study has reported that a force of about 43 kg is needed to rupture the RCL of the index MCP joint.^[3]^ This can be attributed to a great degree of stabilization from the adjacent digits and radial support from the first dorsal interosseous muscle.^[4,5]^ In addition, the RCL is much thicker, wider, stronger, and more oblique in its long axis when compared with the ulnar collateral ligament of the index finger.^[6–8]^ Rapid identification and treatment of patients with unstable index MCP joints is important to avoid progressive instability. Clinical findings suggest that physical examination^[9]^ may play a greater role than imaging studies in diagnosing these injuries. However, on imaging, especially magnetic resonance imaging (MRI) arthrogram, complete tear is seen as a gap on the sequential images.^[10,11]^

These injuries usually occur as the result of the application of an adduction, abduction during a fall onto the outstretched hand, or twist force to a digit.^[7,12]^ However, some patients do not recollect the injury mechanism.^[8,9]^ We report a case of a patient who could not recall the mechanism of injury and presented with bilateral RCL injury. Therefore, we put forth the hypothesis that the injury is thought to be related to her job as a shoemaker. To the best of our knowledge, a similar report has not been presented in the literature so far.

## 2 Case description

**Figure 1:**
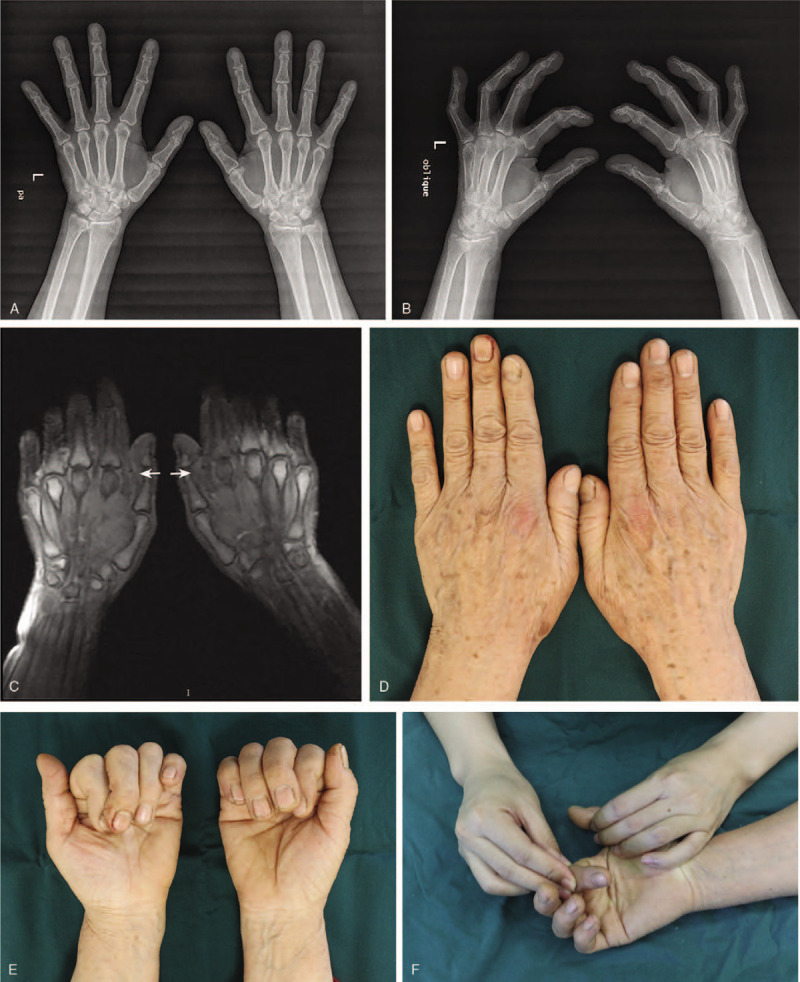
Preoperative imaging. (A and B) MCP joint subluxation of bilateral index fingers on an X-ray. (C) An MRI image confirmed rupture of both RCLs (white arrow). (D) Redness and swelling of the skin at the site of injury. (E) Ulnar deviation and pronation of index finger during active gripping. (F) Passive physical examination. MCP = metacarpophalangeal joint, MRI = magnetic resonance imaging, RCL = radial collateral ligament.

**Figure 2:**
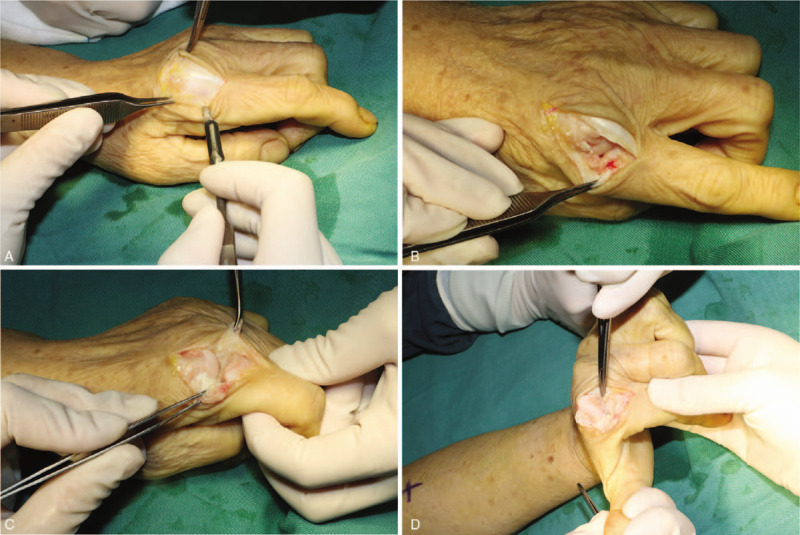
Intraoperative images. (A) An arc manner skin incision on the dorsal radial side of MCP joints. (B) Mid-substance rupture of RCL on both sides. (C and D) Comminution of the base of the proximal phalanx and damage to the articular cartilage.

**Figure 3:**
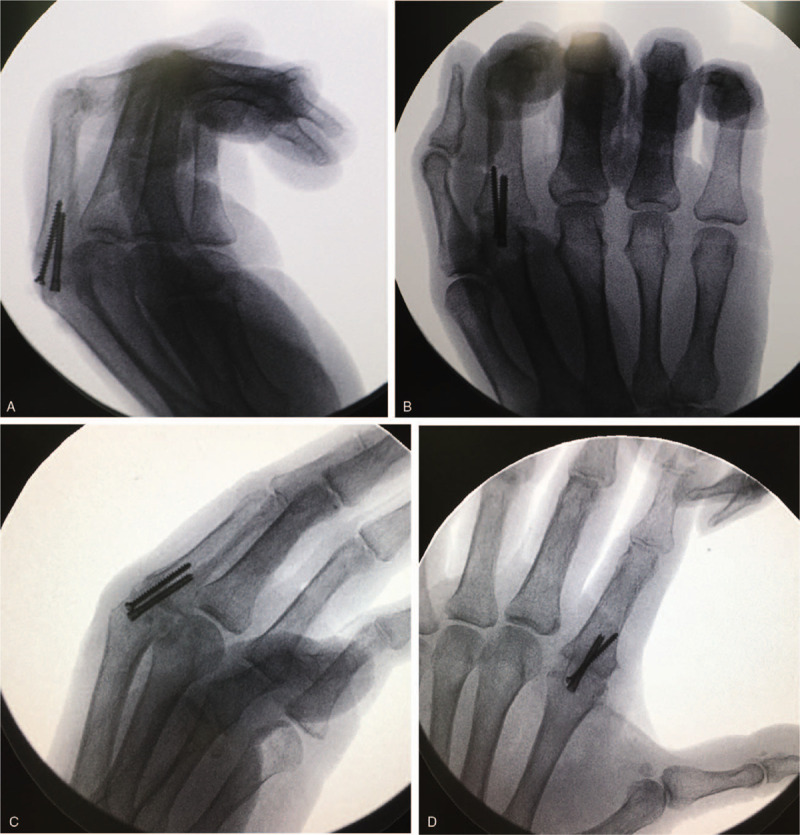
MCP joints were immobilized with screws in 30° of flexion. (A and B) Lateral and anteroposterior X-rays of the right hand. (C and D) Lateral and anteroposterior X-rays of the left hand.

A 58-year-old female was admitted to our department with chief complaints of swelling and pain in the radial aspect of the MCP joint of the bilateral index fingers for 2 years. She did not have any family history of RCL injury. The persistent pain was aggravated while gripping, pulling, buttoning, and twisting, and Bohler's sign was positive on both sides. An X-ray confirmed MCP joint subluxation of both index fingers (Fig. 1A and B). Ultrasonic and MRI examination showed disruption of bilateral RCLs, bone erosion and synovitis of index fingers (Fig. 1C). Physical examination revealed laxity of the RCL without endpoint, slight ulnar deviation and pronation of the index fingers (Fig. 1D–F). Muscle strength was measured using the E-LINK system and a calibrated dynamometer. The average key pinch strength was 5.6 kg in the left hand and 6.1 kg in the right hand, three-jaw strength was 3.6 kg in the left hand and 3.2 kg in the right hand, and tip to tip strength was 1.0 kg in the left hand and 1.4 kg in the right hand. Finally, the patient was diagnosed with RCL injury of both index fingers. During the operation under local anesthesia, we observed second MCP joint subluxation, avulsed RCL in its mid-substance with irregular ends, comminuted fracture at the base of the proximal phalanx, and articular cartilage wear (Fig. 2). After debridement, the MCP joints were immobilized in 25° of flexion and neutral rotation, and the patient was placed in a plaster cast for 6 weeks. The postoperative radiograph showed improvement (Fig. 3), the patient was discharged.

**Figure 4:**
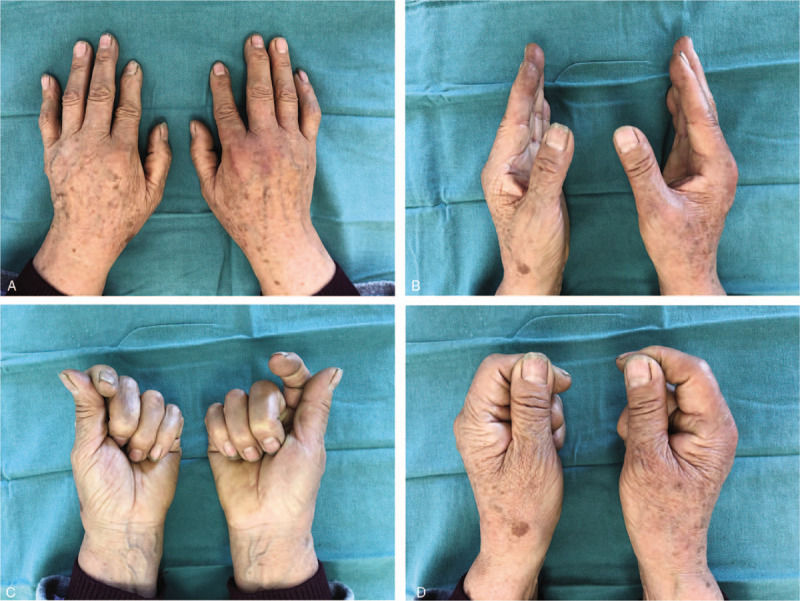
Final Follow-up Images. (A–C) The patient's MCP joints were immobilized in flexion. (D) The patient was able to achieve the key pinch position.

Rehabilitation was initiated by a hand therapist after 6 weeks post-surgery. After the 1-year follow-up, the patient was able to perform her normal daily activities and she did not develop any complications (Fig. 4). The key pinch strength was 5.6 kg in the left hand compared to 6.8 kg in the right hand, three-jaw strength was 4.7 kg in the left hand and 4.0 kg in the right hand, tip to tip strength was 2.7 kg in the left hand and 1.9 kg in the right hand. The patient returned to a full level of activity as a shoemaker.

## 3 Discussion

RCL tears of the index MCP joint can occur at the metacarpal head, the proximal phalanx, or the mid-substance in descending order of frequency.^[13]^ Published researches on RCL injury of the index MCP joint show a unilateral injury.^[7–10,12–16]^ In our study, we reported a case of a 58-year-old woman who presented with chronic bilateral RCL injury of the index MCP joints, with both tears in the mid-substance portion of the RCL.

Gaston et al^[8]^ have reported different grades of injury. Kang et al^[13]^ have reported that the patients can be categorized into the early stage (≤6 weeks) or the late stage (>6 weeks). Based on Gaston and Kang's classification, our patient was categorized into chronic grade 3 type of injury. The specific treatment is variable, and it is based on the grading system. If there is sufficient remnant tissue, primary ligament repair is preferred, and it can be achieved by performing direct suturing or using suture anchors if an avulsion injury occurred.^[17]^ Grade I and II ligament injuries, which present with no joint laxity or laxity with a firm endpoint, can be treated nonoperatively with joint immobilization in 30° to 50° of flexion for 3 to 6 weeks.^[4,8]^ The treatment for grade III injury, where there is no endpoint, is not very well defined in the fingers.^[9]^ Most of the authors recommend surgical intervention, especially for the RCL of the index finger or in the setting of significant joint instability.^[8,13,14]^ We obtained information from the published articles, and very few articles have reported joint fusion (Table 1). But if there is severe arthrosis, joint fusion is appropriate.^[8]^ Considering our patient's age, significant joint instability, and arthrosis, we decided to perform joint fusion.

**Table 1:**
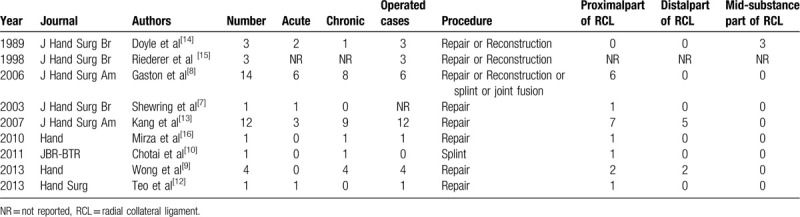
Description of 40 cases of radial collateral ligament injury of index finger MCP joints.

Roquelaure et al^[18]^ reported a case of hand–wrist tendinitis in a worker at a shoe factory. Occupations like shoemaking that involve repetitive work are known to be associated with high incidence of musculoskeletal disorders.^[19]^ The stable pinch mechanism depends on the integrity of the RCL of the index MCP joint combined with the ulnar collateral ligament of the thumb MCP joint.^[10]^ Gupta et al^[20]^ reported reduced pinch strength among shoe factory workers, and they explained that a sore thumb prevented them from exerting maximum pressure. However, the cause of pain was not described. In published articles, the cause of RCL injury of the index finger is mostly traumatic, however, the other causes of RCL injury are not yet known. In our case, the patient presented with long-term symptoms without any history of an obvious accident and she could not recall any reason for this injury. On the basis of the information provided by Roquelaure,^[18]^ we have a high degree of suspicion that this injury resulted from long-term chronic abrasion related to her job as a shoemaker and some amount of pain, as reported by Gupta,^[20]^ resulted from collateral ligament injury.

Finally, injury to the RCL of the index finger is a rare but clinically significant injury due to its effect on functionality of the active limb. A high level of clinical suspicion is needed to diagnose this injury, especially in patients who have specialized jobs that are mainly related to handcraft.

## Acknowledgments

The authors would like to thank nurse Chunjie Liu, Department of hand surgery, China-Japan union hospital, for assistance with the patient's physical information.

## Author contributions

**Conceptualization:** Zhan Zhang, Xiaosong Gu.

**Data curation:** Yueying Li.

**Formal analysis:** Yueying Li.

**Funding acquisition:** Shusen Cui.

**Investigation:** Yueying Li, Guangzhi Wu, Zhan Zhang, Xiaosong Gu.

**Methodology:** Yueying Li, Guangzhi Wu.

**Resources:** Yueying Li, Shusen Cui.

**Supervision:** Zhan Zhang, Xiaosong Gu.

**Writing – original draft:** Yueying Li.

**Writing – review & editing:** Zhan Zhang, Xiaosong Gu.
